# Feasibility and Safety of Transthoracic Echocardiography–Guided Transvenous Permanent Pacemaker Implantation Without Fluoroscopy

**DOI:** 10.1016/j.jacadv.2026.103301

**Published:** 2026-10-05

**Authors:** Tianjie Feng, Ziping Li, Jie Dong, Guangzhi Zhao, Cheng Wang, Jing Dong, Xiangbin Pan

**Affiliations:** Department of Structural Heart Disease, Fuwai Hospital & National Center for Cardiovascular Disease, State Key Laboratory of Cardiovascular Disease, Chinese Academy of Medical Sciences & Peking Union Medical College, National Clinical Research Center for Cardiovascular Diseases, Beijing, China

**Keywords:** fluoroscopy-free, permanent pacemaker, radiation-free implantation, transthoracic echocardiography, TTE-defined lead positioning

## Abstract

**Background:**

Fluoroscopy is routinely used for transvenous permanent pacemaker implantation, but a standardized transthoracic echocardiography (TTE)-only workflow has not been prospectively evaluated.

**Objectives:**

The objective of the study was to evaluate the feasibility, acute safety, and procedural performance of fluoroscopy-free transvenous pacemaker implantation guided exclusively by TTE.

**Methods:**

In this prospective, single-center feasibility study (RADIANCE [Radiation-free Anatomical Direct Imaging Approach for No-fluoroscopy Cardiac Electrode Implantation]; NCT07458724), 52 consecutive patients referred to the Department of Structural Heart Disease were screened. Thirty-one selected patients (59.6%) with guideline-based pacing indications and adequate prespecified supine TTE acoustic windows underwent attempted TTE-only implantation. Acute procedural success required completion without fluoroscopic conversion and fulfillment of prespecified TTE-defined anatomical, electrical, and safety criteria.

**Results:**

TTE-only implantation was successful in 30/31 patients (96.8%; 95% CI: 83.3-99.9), with fluoroscopic conversion in 1/31 (3.2%). The mean procedure time was 55.4 ± 5.6 minutes. No major acute procedural complications occurred. During a median follow-up of 8.8 months (IQR: 6-12 months), atrial lead dislodgement requiring revision occurred in 1/21 dual-chamber recipients (4.8%; 95% CI: 0.1-23.8). Procedure time decreased with chronological case order (−0.59 minutes/case; Newey-West 95% CI: −0.68 to −0.49), an exploratory team-level association.

**Conclusions:**

In selected patients with adequate supine acoustic windows, TTE-guided fluoroscopy-free pacemaker implantation was feasible with acceptable acute outcomes. The lead-position findings and exploratory case-order association require further validation in larger and more diverse populations.

The global use of permanent pacemakers continues to expand in parallel with population aging and broadening indications for device-based bradycardia therapy.^1^^,^^2^ Contemporary registry data estimate that more than 1 million permanent pacemakers are implanted annually worldwide,^1^^,^^3^ underscoring the central role of transvenous pacing in modern clinical electrophysiology. As one of the most frequently performed procedures in cardiovascular medicine, pacemaker implantation represents a substantial and growing component of global health care delivery.

Conventional transvenous pacemaker implantation relies on fluoroscopic guidance^4^ to facilitate venous access, intracardiac navigation, and myocardial lead positioning. Although fluoroscopy provides reliable projectional visualization, it exposes both patients and operators to ionizing radiation.^5^, ^6^, ^7^ Cumulative exposure has been associated with malignancy risk, cataract formation, and orthopedic strain related to chronic lead shielding. In addition, fluoroscopy-dependent workflows require specialized infrastructure and radiation safety systems, which may increase procedural cost and limit accessibility in resource-constrained settings. These concerns have led to increasing emphasis on radiation stewardship in cardiovascular intervention.

Radiation-minimization strategies, including electroanatomical mapping and echocardiographic guidance, have been explored for cardiac device implantation; however, these approaches differ technically from the present transthoracic echocardiography (TTE)-only workflow. Consequently, conventional transvenous pacemaker implantation continues to rely predominantly on fluoroscopy for lead navigation and confirmation of lead position.^8^, ^9^, ^10^, ^11^, ^12^, ^13^, ^14^

Successful transvenous lead implantation requires stable myocardial engagement and satisfactory electrical performance. Although the right atrial appendage is the conventional target for atrial lead placement, the interatrial septum was selected in this feasibility protocol because it could be reproducibly visualized by TTE and provided a consistent target for active-fixation lead deployment.^14^^,^^15^

We therefore prospectively evaluated the feasibility of transvenous permanent pacemaker implantation performed exclusively under TTE guidance, focusing on TTE-defined anatomical outcomes, electrical performance, acute procedural safety, and the exploratory association between case order and team-level procedural performance.

## Methods

### Study design and population

RADIANCE (Radiation-free Anatomical Direct Imaging Approach for No-fluoroscopy Cardiac Electrode Implantation) was a prospective, single-center, single-arm feasibility study conducted in the Department of Structural Heart Disease at Fuwai Hospital. The study is registered at ClinicalTrials.gov (NCT07458724).

Between September 2024 and September 2025, 52 consecutive adults referred to the Department of Structural Heart Disease for de novo transvenous permanent pacemaker implantation were screened. Inclusion required: 1) a guideline-based Class I or IIa pacing indication; 2) aged 18 to 80 years; and 3) intrinsic QRS duration <120 ms. Four patients with QRS duration ≥120 ms or a cardiac resynchronization therapy indication did not meet the inclusion criterion. For baseline indication classification, atrial fibrillation (AF) required symptomatic slow ventricular response and/or prolonged ventricular pauses after reversible causes had been excluded.^16^

After inclusion assessment, patients were excluded for: 1) failure to meet mandatory supine TTE-window criteria; 2) hemodynamic instability requiring emergent temporary pacing; 3) left ventricular ejection fraction <35% with an indication for an alternative device strategy; 4) severe tricuspid regurgitation (TR) (grade ≥3); 5) right-heart anatomy precluding safe TTE-guided septal targeting, including complex congenital malformation or prior repair distorting septal anatomy, prior tricuspid intervention, an intracardiac device involving the intended target, or marked right-sided chamber deformation; or 6) an indication for nontransvenous or epicardial pacing.

The department-specific screening and enrollment flow is shown in Figure 1.

**Figure 1:**
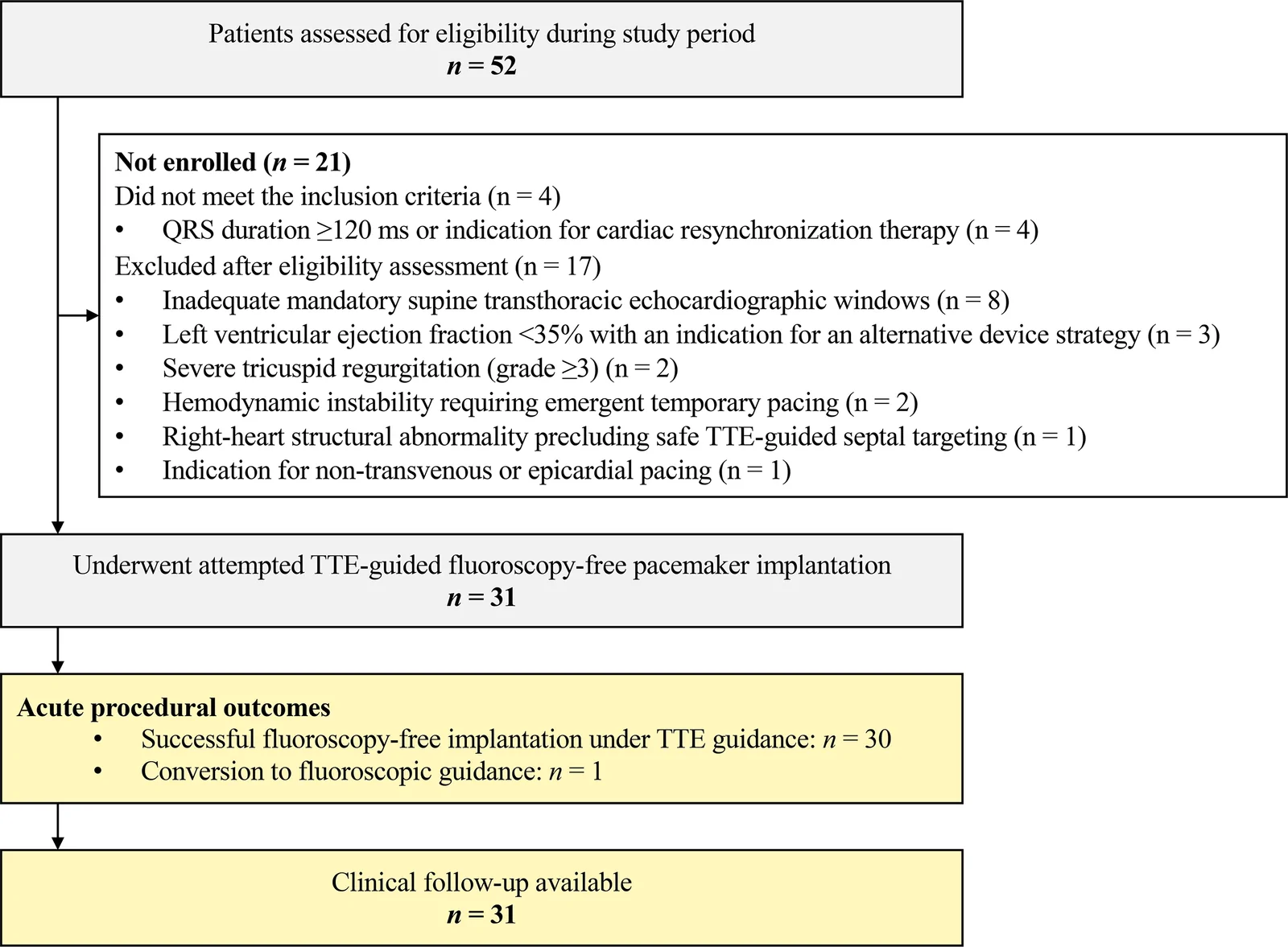
Patient Screening and Procedural Outcomes Among 52 consecutive patients referred to the Department of Structural Heart Disease, 4 did not meet the QRS-duration inclusion criterion and 17 met an exclusion criterion. Thirty-one underwent attempted TTE-only implantation; 30 were completed without fluoroscopy and 1 required fluoroscopic conversion. Follow-up was available for all 31 patients. TTE = transthoracic echocardiography.

### Preoperative evaluation and data adjudication

Acoustic-window eligibility was assessed twice with the patient supine: before transfer to the procedure room and again immediately before sterile preparation. Mandatory acoustic-window criteria were prespecified in the study protocol before enrollment of the first participant^17^: 1) the superior vena cava (SVC)-right atrium (RA) junction in a subcostal bicaval or modified subcostal view; 2) the RA, tricuspid valve, and right ventricle (RV) in an apical four-chamber (A4C) or RV inflow view; 3) the interventricular septum and RV free wall in at least 2 orthogonal planes incorporating parasternal long- and short-axis views; and, for planned dual-chamber implantation, 4) the interatrial septum in both apical and subcostal four-chamber views. A window was inadequate when the target could not be continuously distinguished during a respiratory cycle or orthogonal confirmation was not reproducible after optimization. All mandatory views were required to be assessed during each procedure, but their sequence, repetition, and duration were adapted to the procedural step and individual anatomy; view-specific utilization time was not prospectively recorded.

Vascular ultrasound assessed the accessible right axillary and extrathoracic subclavian veins. Although intrathoracic or central venous obstruction could not be excluded, patients with suggestive findings—including upper-extremity or cervicofacial swelling, dilated venous collaterals, or unexplained dyspnea—were excluded.^18^ Failure of guidewire advancement along the expected central venous course was a prespecified indication for rescue fluoroscopy or an alternative access strategy.

Baseline and follow-up echocardiographic measurements were independently performed by 2 experienced sonographers and reviewed according to a predefined standardized imaging protocol. In cases of discrepancy, consensus was reached through joint review. This process was distinct from a formal interobserver reproducibility assessment of intraprocedural views, which was not prospectively performed. Clinical and procedural data were independently extracted by 2 cardiothoracic surgeons from the institutional database and procedural reports and cross-checked for accuracy.

The study protocol was approved by the institutional ethics committee. All procedures complied with the Declaration of Helsinki, and all participants provided written informed consent.

Figures 2A to 2C shows the operating room layout, antisepsis field, and final draping. Antisepsis covered the lower anterior neck, bilateral supraclavicular and clavicular regions, shoulders, proximal upper arms, and broad anterior chest. Overlapping sterile drapes created a single right upper-chest operative window. The transducer and cable were enclosed in a continuous sterile cover and introduced beneath an overlapping drape edge, without a second fenestration, to access the subcostal, apical, and parasternal windows. Transducer movement was strictly confined away from the right-sided operative field; when necessary, an assistant maintained a boundary with a hand or positioning aid placed outside the sterile field. Depth, gain, focus, and sector width were optimized in real time.

**Figure 2:**
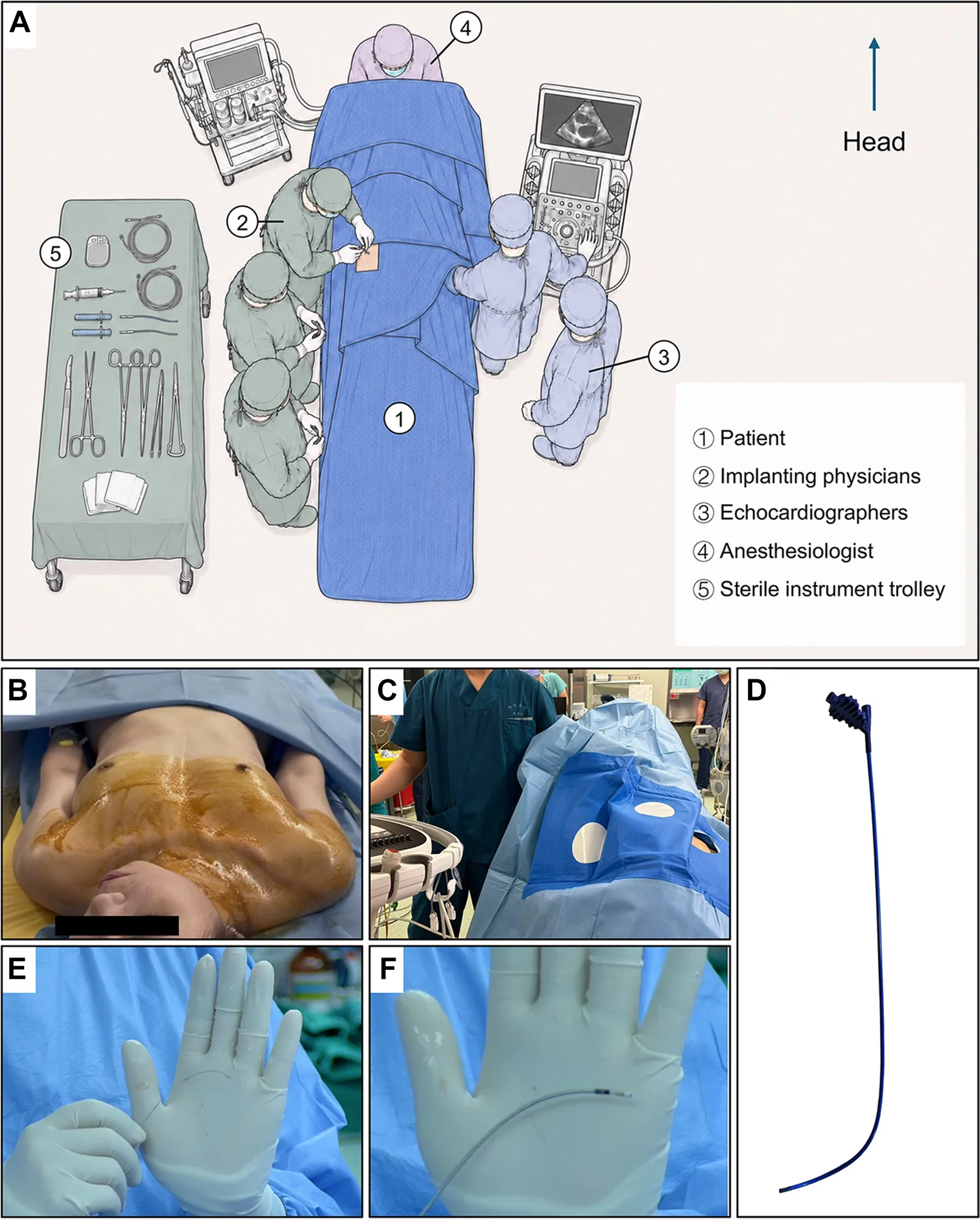
Operating Room Preparation and Lead Delivery Tools (A) Operating-room layout. (B) Antisepsis of the lower anterior neck, bilateral supraclavicular/clavicular regions, shoulders, proximal upper arms, and broad anterior chest. (C) Final overlapping sterile drapes, right upper-chest operative window, and echocardiographer/ultrasound-console relationship; probe access is beneath a drape edge without a second fenestration. (D) Custom fixed-curve slittable delivery sheath developed by the study team to provide backup support and gross orientation from the SVC toward the interatrial septum. (E) Manual preshaping of the atrial lead stylet for distal steering. (F) Resulting distal lead/stylet curvature used to adjust lead-tip direction and contact angle.

### Procedure

All procedures were performed by the same core multidisciplinary team: 3 implanting physicians at the patient's right side, the same experienced primary echocardiographer acquiring images from the left/head side, and a second echocardiographer present for real-time on-site review. The physicians had experience in conventional transvenous implantation or structural-intervention imaging, but no prior clinical series using this TTE-only workflow. Fluoroscopy and temporary pacing were immediately available for rescue.

All procedures used a protocol-specified right-sided venous approach under local anesthesia, with conscious sedation when clinically indicated. The right side provided a relatively short path from the axillary/extrathoracic subclavian vein to the SVC and left the left parasternal and apical chest wall accessible to the echocardiographer; this workflow choice does not imply general superiority over conventional left-sided implantation. Ultrasound-guided venous access and guidewire entry into the RA were confirmed using supraclavicular and subcostal bicaval views (Figures 3A and 3B).

**Figure 3:**
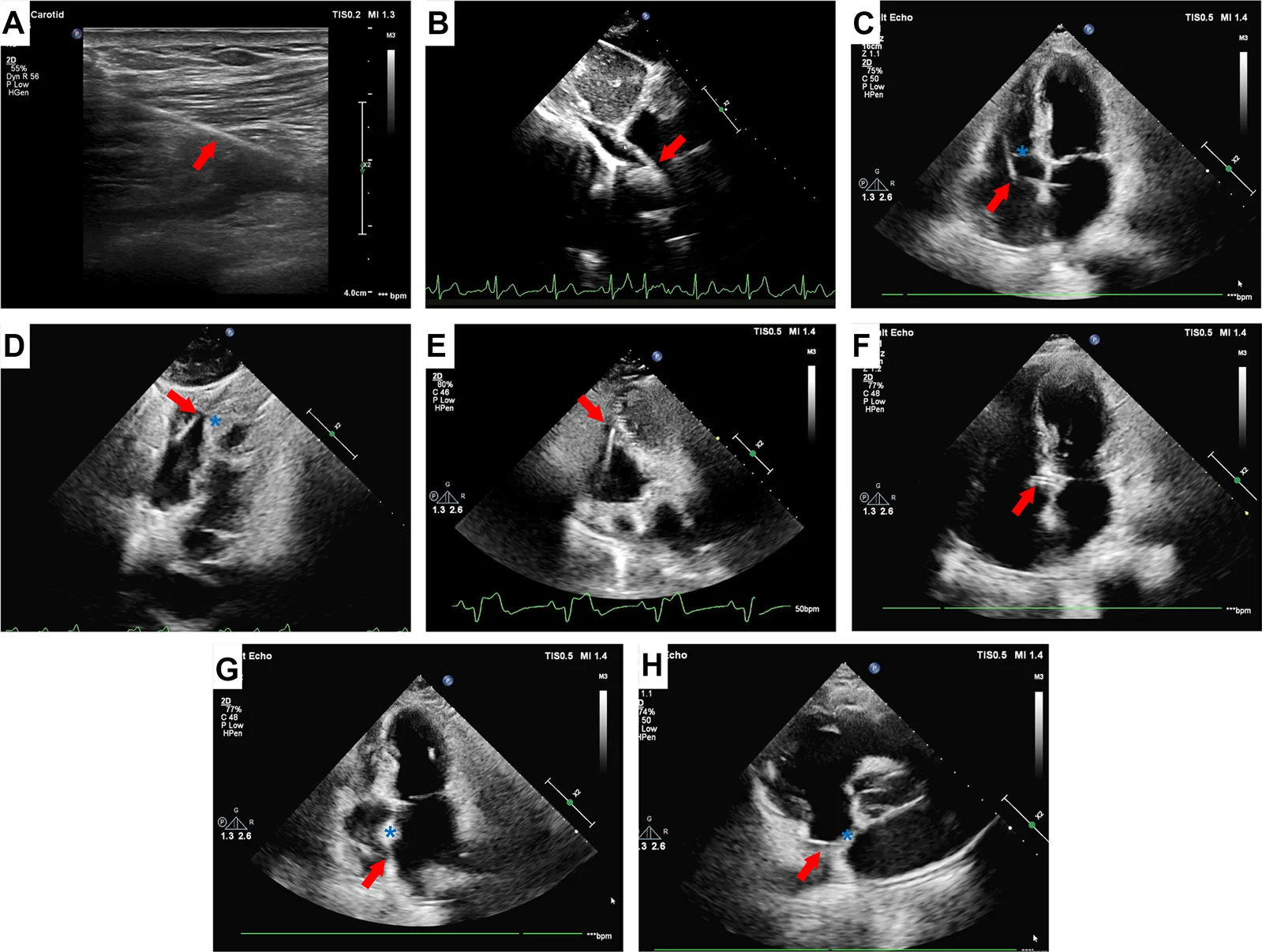
Transthoracic Echocardiography–Guided Lead Implantation Sequence (A) Guidewire in the accessible right axillary/extrathoracic subclavian vein. (B) Subcostal bicaval view of guidewire passage through the superior vena cava into the right atrium. (C) Ventricular lead crossing the tricuspid valve. (D) Ventricular lead directed toward the interventricular septum in an orthogonal view. (E) Final image meeting the study-defined transthoracic echocardiography criteria for apparent ventricular septal apposition and the lead configuration used with respiratory, extracardiac, electrical, and tricuspid regurgitation criteria for slack assessment. (F) Double-track sign of the atrial delivery sheath directed toward the interatrial septum. (G) Atrial lead emerging from the sheath toward the interatrial septum. (H) Parasternal short-axis assessment of atrial lead orientation. Red arrows indicate the guidewire in A and B, the ventricular lead in C to E, the atrial delivery sheath in F, and the atrial lead in G and H. Blue asterisks indicate the tricuspid valve in C, the interventricular septal target in D, and the interatrial septal target in G and H.

An active-fixation ventricular lead was advanced through a slittable sheath under sequential A4C, RV inflow, parasternal long-axis, and parasternal short-axis imaging. A4C and RV inflow views were used for tricuspid crossing; orthogonal parasternal views assessed ventricular septal direction and excluded an RV free-wall trajectory. Figures 3C to 3E shows the continuous sequence from tricuspid crossing to final TTE-defined septal apposition.

Final TTE-defined ventricular septal apposition required concordant findings in at least 2 orthogonal planes, persistent contact without recoil during gentle push-pull testing, respiratory stability, and acceptable bipolar and unipolar sensing, pacing threshold, impedance, and injury current. TTE cannot directly visualize the fixation helix or prove the complete 3-dimensional lead course; therefore, no claim of independently validated true septal position is made.

The right atrial appendage is the conventional atrial target but was not reliably delineated by TTE because of its anterior near-field location, adjacent lung/ribs and epicardial fat, and reverberation artifacts. The protocol therefore used the interatrial septum as a study-defined TTE target that could be visualized in the required views. A custom fixed-curve slittable delivery sheath with a dilator, developed by our team, was used for atrial lead delivery (Figure 2D). Its distal curve was designed to follow the transition from the SVC into the RA and to provide backup support and gross orientation toward the interatrial septum.

Manual preshaping of the atrial lead stylet served a complementary function: after the distal lead segment emerged from the sheath, the stylet curve permitted finer adjustment of lead-tip direction and contact angle according to individual right atrial anatomy (Figures 2E and 2F). After fixation, the sheath was removed using its slittable design. A4C and subcostal four-chamber views assessed medial direction and the double-track sign; parasternal short-axis imaging was used to exclude an apparent posterior course toward the coronary sinus (Figures 3F to 3H). Suspected coronary-sinus entry required withdrawal and repositioning before helix deployment.

TTE surveillance was maintained throughout. Color Doppler TR was assessed before and after each slack adjustment and again at final inspection. Slack was determined by combined criteria: respiratory stability of the lead tip; absence of a taut straight course or excessive tricuspid-level redundancy; sufficient extracardiac length retained at the pocket because TTE cannot show the entire lead; and stable sensing, threshold, and impedance before and after adjustment. Figure 3E shows the representative final ventricular lead configuration used with these criteria. After sheath removal and generator connection, multiplane TTE confirmed unchanged TTE-defined lead position, no new pericardial effusion, and no significant increase in TR. Videos 1 and 2 show the complete stepwise workflow in 2 representative cases and demonstrate interpatient variability in obtainable views.

### Study endpoints

The primary endpoint was acute procedural success, defined as completion under TTE guidance without fluoroscopic conversion and fulfillment of prespecified TTE-defined anatomical, electrical, and safety criteria. The ventricular TTE-defined anatomical criterion required persistent apparent septal apposition after sheath withdrawal and slack adjustment in at least 2 orthogonal planes, without an apparent RV free-wall trajectory. Dual-chamber cases additionally required a TTE-defined interatrial-septal orientation and exclusion of apparent coronary-sinus entry. Coronary-sinus helix deployment was classified as failure of the protocol-defined anatomical criterion and a major safety event. Electrical and major-complication thresholds remained unchanged from the prespecified protocol.

Secondary endpoints included procedure duration, venous-access, and lead-positioning times, TTE-guidance time, acute electrical performance, and 3-month device stability. Follow-up safety included lead revision, thromboembolic events, new AF, TR progression, pericardial effusion, infection, and mortality.

Safety endpoints during follow-up included major adverse events, structural complications detected by serial TTE, and lead-related adverse events requiring intervention. Follow-up duration was calculated from the implantation date to the most recent documented clinical visit or device interrogation. For event analyses, patients without the event of interest were censored on the date of their last available follow-up.

### Statistical analysis

Continuous variables are presented as mean ± SD or median with IQR, according to distribution assessed by the Shapiro-Wilk test. Categorical variables are reported as counts and percentages.

Chronological case order was analyzed as an exploratory team-level workflow measure rather than an individual-operator learning curve. Ordinary least-squares linear regression was used to estimate the change in procedure time per case. Because observations were ordered chronologically and residual serial correlation could not be excluded, uncertainty for the slope was estimated using Newey-West heteroskedasticity- and autocorrelation-consistent SEs with lag 1. The conventional model-based interval and a bias-corrected and accelerated bootstrap interval based on 5,000 resamples were calculated as sensitivity analyses. Segmented regression and cumulative sum (CUSUM) analyses were descriptive and hypothesis-generating and were not used to define competency or a generalizable training threshold.

Exploratory segmented regression was used to describe possible nonlinearity beyond the unsegmented linear model. Candidate breakpoints from cases 5 through 26 were evaluated, ensuring at least 5 observations on each side, and the breakpoint minimizing the residual sum of squares was selected. The model was parameterized as follows:

$$Yᵢ=\beta_{0}+\beta_{1}\left(i-1\right)+\beta_{2}I\left(i\;>\;\psi \right)+\beta_{3}\left(i-\psi \right)I\left(i\;>\;\psi \right)+\varepsilon ᵢ$$

where i denotes chronological case order, ψ the selected breakpoint, and I the indicator function. β_0_ represents the predicted procedure time at case 1, β_1_ the prebreakpoint slope, β_2_ the estimated discontinuity between the fitted segments at the breakpoint, and β_3_ the change in slope; the postbreakpoint slope is β_1_ + β_3_. CIs were conditional on the selected breakpoint and did not account for breakpoint-selection uncertainty. The analysis was descriptive and hypothesis-generating and was not interpreted as evidence of skill acquisition or procedural mastery.

CUSUM analyses were performed according to established surgical performance frameworks.^19^^,^^20^ For the binary outcome, the CUSUM statistic was calculated as the CUSUM of observed failure minus 0.10. This value represented a descriptive reference corresponding to 90% procedural success; it was not derived from the study data, supported as an external performance benchmark, or treated as a formal acceptable failure rate. For procedure duration, the CUSUM statistic was calculated as the cumulative deviation of each procedure time from the overall mean. No unacceptable failure rate or decision limits were prespecified; therefore, both CUSUM plots were interpreted descriptively and were not considered evidence of statistical process control, competency, or sustained performance improvement.

Exact 2-sided Clopper-Pearson 95% CIs were calculated for key binomial outcomes. For descriptive comparison, the early and late phases were defined as cases 1 to 15 and cases 16 to 31, respectively. Atrial-specific variables were not applicable to the 10 single-chamber cases and were summarized among the 21 dual-chamber recipients; no other baseline, procedural, or endpoint data were missing, and no imputation was performed. All statistical tests were 2-sided, and *P* < 0.05 was considered statistically significant. Analyses were performed using StataNow (version 19.5, StataCorp) and R (version 4.5.1, R Foundation for Statistical Computing).

## Results

### Baseline characteristics

Of the 52 consecutive department-referred patients screened, 4/52 (7.7%) did not meet the QRS-duration inclusion criterion and 17/52 (32.7%) met an exclusion criterion; 31/52 (59.6%) underwent attempted TTE-only implantation (Figure 1). All 31/31 enrolled patients met the mandatory acoustic-window criteria at both supine preprocedural assessments. The 8 patients excluded because of inadequate mandatory acoustic windows had a mean body mass index of 24.5 ± 3.8 kg/m^2^. The enrolled patients had a mean age of 64.8 ± 7.7 years, 20/31 (64.5%) were males, and a mean body mass index of 24.0 ± 2.8 kg/m^2^ (Table 1).

**Table 1:** Baseline Clinical and Device Characteristics (N = 31)

Demographics	
Age, y	64.8 ± 7.7
Male, n (%)	20 (64.5)
BMI, kg/m^2^	24.0 ± 2.8
Indication for implantation	
Sinus node dysfunction, n (%)	15 (48.4)
High-grade AV block^a^, n (%)	7 (22.6)
Atrial fibrillation with symptomatic slow ventricular response and/or prolonged ventricular pauses, n (%)	9 (29.0)
Baseline ECG parameters	
Intrinsic heart rate, beats/min	52.4 ± 5.2
Minimum heart rate (Holter), beats/min	38.5 ± 5.3
QRS duration, ms	98.2 ± 12.3
PR interval, ms^b^	185.5 ± 35.7
Echocardiographic parameters	
LVEF, %	62.5 ± 8.1
LVEDD, mm	49.2 ± 4.6
Structural heart disease, n (%)	4 (12.9)
Comorbidities	
Hypertension, n (%)	22 (71.0)
Coronary artery disease, n (%)	4 (12.9)
Cerebrovascular disease, n (%)	4 (12.9)
Chronic kidney disease, n (%)	2 (6.5)
Paroxysmal AF, n (%)	5 (16.1)
Device type	
Dual-chamber pacemaker, n (%)	21 (67.7)
Single-chamber pacemaker, n (%)	10 (32.3)

Continuous variables are presented as mean ± SD or, median (IQR), according to distribution assessed by the Shapiro-Wilk test. Categorical variables are reported as n (%).

AF = atrial fibrillation; AV = atrioventricular; BMI = body mass index; ECG = electrocardiogram; LVEDD = left ventricular end-diastolic diameter; LVEF = left ventricular ejection fraction.

a High-grade atrioventricular block includes second-degree type II and complete atrioventricular block.

b PR interval was assessed only in patients in sinus rhythm at baseline; PR measurement is not applicable in atrial fibrillation.

Pacing indications were sinus node dysfunction in 15/31 patients (48.4%), high-grade atrioventricular block in 7/31 (22.6%), and AF with symptomatic slow ventricular response and/or prolonged ventricular pauses in 9/31 (29.0%) (Table 1).

Baseline intrinsic heart rate averaged 52.4 ± 5.2 beats per minute, with a minimum heart rate of 38.5 ± 5.3 beats per minute on ambulatory monitoring. The mean QRS duration was 98.2 ± 12.3 ms, and the PR interval among patients in sinus rhythm was 185.5 ± 35.7 ms. Left ventricular ejection fraction was preserved (62.5 ± 8.1%), and mean left ventricular end-diastolic diameter was 49.2 ± 4.6 mm. Structural heart disease was present in 4/31 patients (12.9%) (Table 1).

Comorbidities included hypertension in 22/31 patients (71.0%), coronary artery disease in 4/31 (12.9%), cerebrovascular disease in 4/31 (12.9%), chronic kidney disease in 2/31 (6.5%), and paroxysmal AF in 5/31 (16.1%) (Table 1).

Regarding device configuration, 21/31 patients (67.7%) received dual-chamber pacemakers and 10/31 (32.3%) received single-chamber ventricular pacemakers. Ventricular leads were directed toward the protocol-defined interventricular-septal TTE target. In dual-chamber systems, atrial leads were directed toward the protocol-defined interatrial-septal TTE target; these descriptions do not constitute independently validated anatomical locations (Table 1).

### Clinical endpoints

Acute procedural success was achieved in 30/31 patients (96.8%; exact 95% CI: 83.3-99.9). Conversion to fluoroscopy was required in 1/31 patient (3.2%; exact 95% CI: 0.1-16.7) because the protocol criterion for stable TTE-defined ventricular septal apposition could not be met under echocardiographic guidance alone (Table 2).

**Table 2:** Procedural Performance and Clinical Outcomes (N = 31)

Primary endpoint	
Acute procedural success, n (%)	30/31 (96.8%; 95% CI: 83.3-99.9)
Conversion to fluoroscopy, n (%)	1/31 (3.2%; 95% CI: 0.1-16.7)
Procedural performance	
Total procedure time, min	55.4 ± 5.6
Venous access time, min	7.5 ± 3.3
Ventricular lead positioning time, min	11.8 ± 3.0
Atrial lead positioning time^a^, min	15.1 ± 3.5
Intraoperative TTE guidance time, min	42.0 ± 5.6
Acute electrical performance	
Ventricular pacing threshold, V	0.81 ± 0.16
Ventricular R-wave sensing, mV	9.7 ± 2.0
Ventricular impedance, Ω	690.8 ± 155.0
Atrial pacing threshold^a^, V	0.82 ± 0.18
Atrial sensing^a^, mV	2.4 ± 0.4
Atrial impedance^a^, Ω	672.3 ± 153.5
Acute safety	
Cardiac perforation, n (%)	0/31 (0%)
Pericardial tamponade, n (%)	0/31 (0%)
Pneumothorax requiring intervention, n (%)	0/31 (0%)
Major vascular injury, n (%)	0/31 (0%)
Pocket hematoma requiring intervention, n (%)	0/31 (0%)
Procedure-related mortality, n (%)	0/31 (0%)
3-month device stability	
Lead dislodgement (any), n (%)	1/31 (3.2%; 95% CI: 0.1-16.7)
Ventricular lead dislodgement, n (%)	0/31 (0%)
Atrial lead dislodgement^a^, n (%)	1/21 (4.8%; 95% CI: 0.1-23.8)
Reintervention required, n (%)	1/31 (3.2%; 95% CI: 0.1-16.7)
Follow-up outcomes	
Median follow-up duration, mo	8.8 (IQR 6-12)
Thromboembolic events, n (%)	0/31 (0%)
New-onset atrial fibrillation, n (%)	0/31 (0%)
Significant TR progression (≥1 grade), n (%)	0/31 (0%)
All-cause mortality, n (%)	0/31 (0%)

TTE = transthoracic echocardiography; TR = tricuspid regurgitation.

a Atrial electrical parameters, atrial lead positioning time, and atrial lead dislodgement are reported among dual-chamber recipients (n = 21). Pacing thresholds were measured at a pulse width of 0.4 ms. Exact Clopper-Pearson 95% CIs are reported for key binomial outcomes. Continuous variables are presented as mean ± SD or median (IQR).

In the 30/30 successful TTE-only cases, multiplane images met the prespecified study criteria for TTE-defined apparent interventricular-septal apposition and, in dual-chamber systems, interatrial-septal orientation. Immediate TTE showed no new pericardial effusion or significant TR worsening.

Procedural performance metrics demonstrated a mean total procedure duration of 55.4 ± 5.6 minutes. The mean venous access time was 7.5 ± 3.3 minutes, ventricular lead positioning time was 11.8 ± 3.0 minutes, and atrial lead positioning time among 21 dual-chamber recipients was 15.1 ± 3.5 minutes. The mean intraoperative TTE guidance time was 42.0 ± 5.6 minutes. Fluoroscopy time was zero in all 30/30 successful TTE-only procedures (Table 2).

All 30/30 successful cases met predefined acute electrical performance criteria. The mean ventricular pacing threshold was 0.81 ± 0.16 V (pulse width 0.4 ms), with R-wave sensing amplitude of 9.7 ± 2.0 mV and impedance of 690.8 ± 155.0 Ω. Among the 21 dual-chamber recipients, the mean atrial pacing threshold was 0.82 ± 0.18 V, P-wave sensing amplitude was 2.4 ± 0.4 mV, and impedance was 672.3 ± 153.5 Ω (Table 2).

No major acute procedural complications occurred. Specifically, there were no cases of cardiac perforation, pericardial tamponade, pneumothorax requiring intervention, major vascular injury, pocket hematoma requiring intervention, or procedure-related mortality (Table 2).

At 3-month follow-up, early device stability was maintained in 30/31 patients (96.8%; exact 95% CI: 83.3-99.9). Atrial lead dislodgement requiring revision occurred in 1/31 patients overall (3.2%; exact 95% CI: 0.1-16.7) and in 1/21 dual-chamber recipients (4.8%; exact 95% CI: 0.1-23.8); ventricular lead dislodgement occurred in 0/31. During a median follow-up of 8.8 months (IQR: 6-12 months), thromboembolic events, new-onset AF, significant TR progression (≥1 grade), and all-cause mortality each occurred in 0/31 patients (Table 2).

### Exploratory case-order analysis

In the exploratory team-level case-order analysis, procedure time decreased by 0.59 minutes per chronological case (Newey-West 95% CI: −0.68 to −0.49; conventional 95% CI: −0.66 to −0.52; bias-corrected and accelerated bootstrap 95% CI: −0.68 to −0.52) (Figure 4). This association describes chronological workflow change within a stable multidisciplinary team but cannot establish an individual-operator learning curve. Exploratory segmented regression selected a breakpoint at case 5. Conditional on this selected breakpoint, the predicted procedure time at case 1 was 68.40 minutes (95% CI: 66.31-70.49), the prebreakpoint slope was −2.10 minutes/case (95% CI: −2.95 to −1.25), the estimated discontinuity at the breakpoint was 0.70 minutes (95% CI: −1.66 to 3.05), and the slope change was 1.58 minutes/case (95% CI: 0.73-2.44), yielding an implied postbreakpoint slope of −0.52 minutes/case (95% CI: −0.59 to −0.45). Mean procedure time was 59.9 ± 3.9 minutes for cases 1 to 15 and 51.2 ± 3.0 minutes for cases 16 to 31. Residual diagnostics for the unsegmented model are shown in Supplemental Figure 1. All case-order analyses were descriptive and hypothesis-generating.

**Figure 4:**
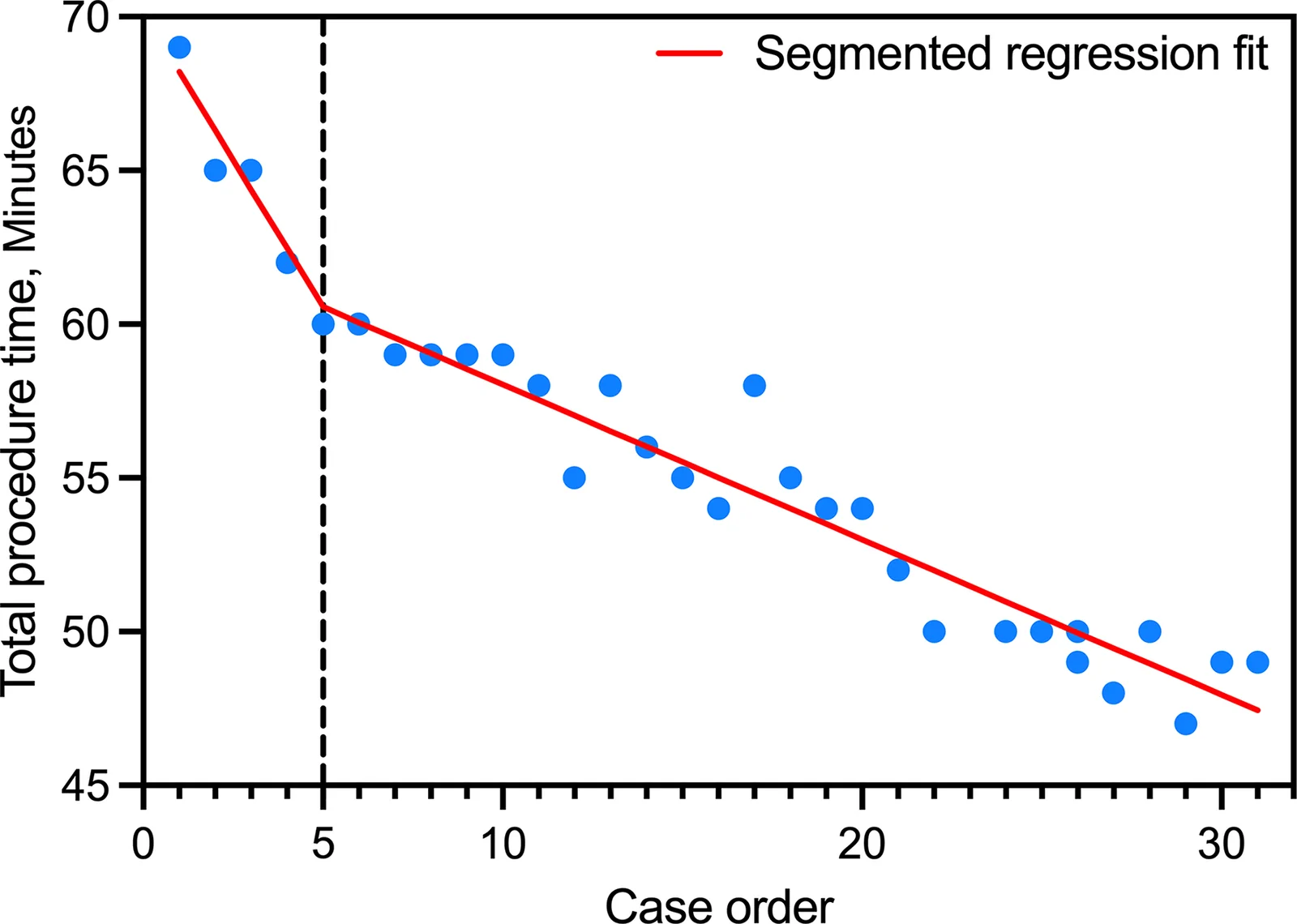
Exploratory Team-Level Case-Order Analysis Total procedure time is plotted by chronological case order. The red line represents an exploratory segmented linear regression fit, and the vertical dashed line marks the selected breakpoint at case 5. In a separate unsegmented linear regression, procedure time decreased by 0.59 minutes per case (Newey-West 95% CI: −0.68 to −0.49). Both analyses are descriptive and hypothesis-generating; they do not define an individual-operator learning curve, competence threshold, or generalizable training requirement.

Exploratory CUSUM plots described chronological patterns in procedural failure and procedure time (Supplemental Figure 2). For the binary CUSUM analysis, deviations were calculated using 0.10 solely as a descriptive reference corresponding to 90% success. The curve showed a transient upward excursion following the single procedural failure and subsequently declined to −2.1 by case 31 (Supplemental Figure 2A). Because 0.10 was not an externally validated performance benchmark and no unacceptable failure rate or decision limits were prespecified, this plot does not provide formal evidence of statistical process control. Overall acute procedural success was 30/31 (96.8%), with no additional failures after the early event.

For the continuous CUSUM analysis, each procedure time was centered on the overall sample mean. The curve initially increased and subsequently declined after approximately cases 15 to 17 (Supplemental Figure 2B), indicating that procedure times in the later chronological cases were more often below the overall sample mean.

## Discussion

In this prospective feasibility study, transvenous pacemaker implantation was completed without fluoroscopy in 30 of 31 selected patients. Acute electrical performance was acceptable, and no major acute procedural complications occurred (Central Illustration). These findings support the feasibility of TTE-guided implantation in selected patients with adequate supine acoustic windows and warrant further evaluation in larger, multicenter studies.

**Central Illustration:**
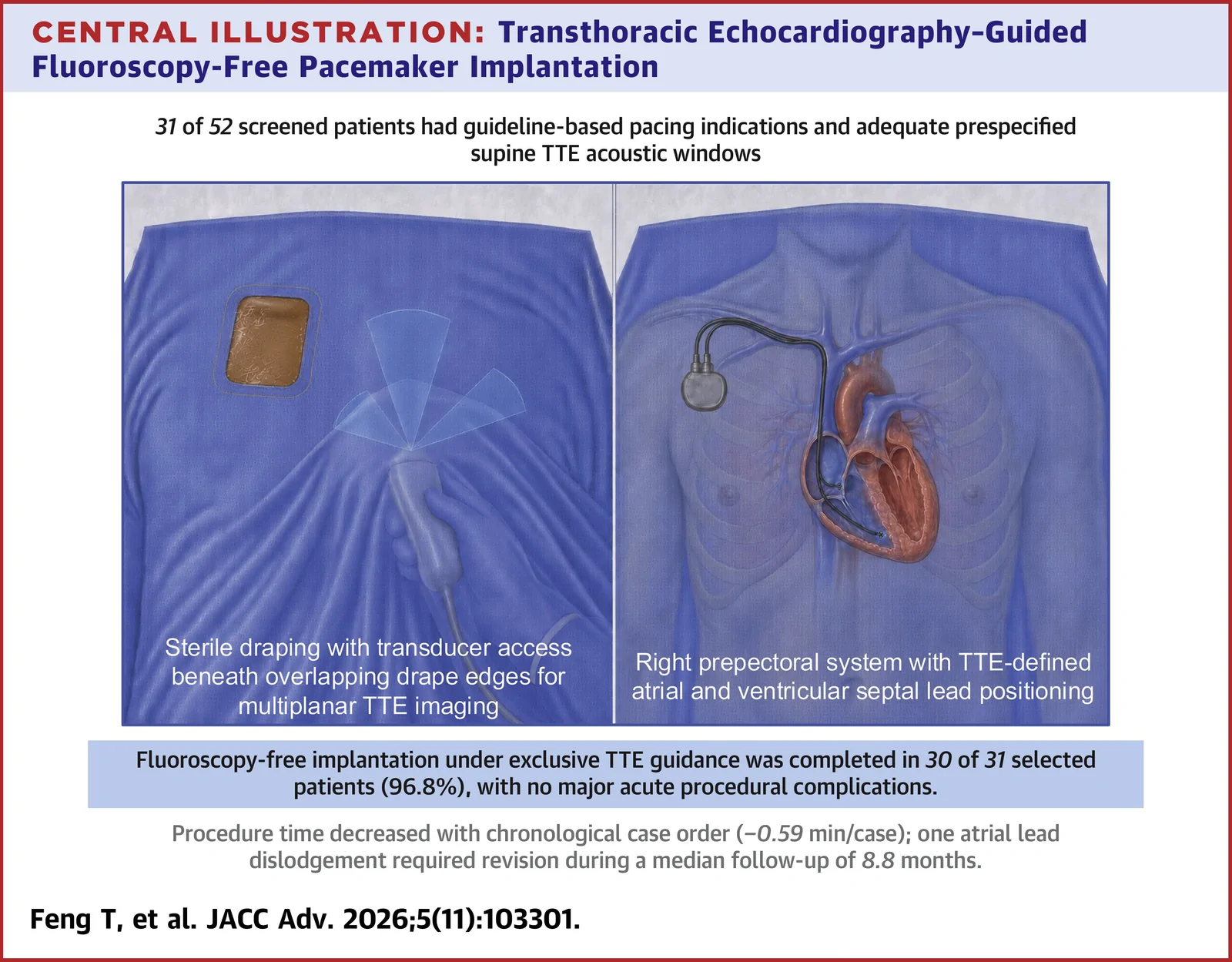
Transthoracic Echocardiography–Guided Fluoroscopy-Free Pacemaker Implantation Among 52 department-referred patients screened, 31 met the clinical and prespecified supine acoustic-window criteria. Sterile transducer access beneath overlapping drape edges enabled multiplane TTE guidance. Implantation was completed without fluoroscopy in 30 of 31 selected patients, with no major acute procedural complications; 1 atrial lead dislodgement required revision during median 8.8-month follow-up. Lead location and slack were assessed using protocol-defined TTE, extracardiac, and electrical criteria and were not independently validated. TTE = transthoracic echocardiography.

### Comparison with existing radiation-sparing approaches

To our knowledge, this is the first prospective feasibility study to evaluate a standardized, fully transthoracic workflow for conventional transvenous pacemaker implantation without intraprocedural fluoroscopic guidance. TTE provided step-specific 2-dimensional structural visualization.

Previous fluoroscopy-sparing pacemaker procedures have followed distinct technical pathways. Three-dimensional electroanatomical mapping systems have enabled transvenous pacemaker implantation without fluoroscopy in selected cases,^8^^,^^9^ particularly in special populations such as pregnant patients,^10^ but require dedicated mapping infrastructure and electrophysiologic expertise. Echocardiographic guidance has also been used during leadless pacemaker implantation, most commonly with transesophageal echocardiogram,^11^ although these procedures involved femoral delivery systems and frequently require sedation or general anesthesia. Isolated reports have described TTE-assisted lead positioning,^12^^,^^13^ including physiological pacing strategies, but a standardized TTE-guided workflow for conventional transvenous permanent pacemaker implantation has not previously been prospectively evaluated.

Radiation-minimization strategies have also been increasingly adopted across cardiovascular interventions. Near-zero or zero-fluoroscopy catheter ablation is increasingly feasible with electroanatomical mapping,^21^, ^22^, ^23^ whereas echocardiographic guidance has reduced fluoroscopic dependence in structural interventions.^24^, ^25^, ^26^, ^27^, ^28^ Ultrasound-guided vascular access has additionally improved safety compared with landmark-based or fluoroscopic techniques in cardiac implantable electronic device implantation.^29^^,^^30^ The present study evaluated a selected TTE-guided workflow for conventional transvenous pacemaker implantation performed under local anesthesia, with conscious sedation when clinically indicated.

### Technical considerations and imaging strategy

TTE-guided pacemaker implantation introduces specific technical challenges. Two-dimensional echocardiography cannot display the entire lead trajectory in a single imaging plane, and some conventional lead targets, particularly the right atrial appendage, may not be reliably visualized. We therefore used systematic multiwindow interrogation to infer, rather than definitively establish, lead orientation relative to the interventricular and interatrial septa.

Protocol-defined septal targeting required assessment of lead orientation, apparent myocardial apposition, and fixation stability. In this study, multiplane TTE was combined with real-time electrical measurements to evaluate TTE-defined orientation and apparent apposition. Electrical parameters confirmed effective myocardial capture and sensing but did not independently verify anatomical lead location. Continuous TTE also enabled real-time surveillance for pericardial effusion, tricuspid-valve interaction, and other structural complications.^31^^,^^32^

### Clinical implications and future directions

Radiation protection should be considered along a spectrum. Suspended personal protection systems such as zero-gravity can substantially reduce operator radiation exposure and orthopedic burden while preserving conventional fluoroscopic guidance.^33^ TTE-guided implantation should therefore be viewed as 1 component of a broader radiation-minimization strategy rather than the sole radiation-sparing option.

Electroanatomical mapping and TTE may be complementary in future workflows. Mapping can provide electrical-spatial navigation, whereas TTE offers direct tissue visualization and continuous surveillance for structural complications. A hybrid approach may mitigate the limitations of either modality alone, although this strategy was not evaluated in the present study.

Extension to conduction-system pacing also warrants further investigation. TTE-guided left bundle branch area pacing has been described,^13^ but confirmation of conduction-system capture requires intracardiac electrograms and paced-QRS criteria in addition to anatomical visualization.^34^^,^^35^ Similarly, Bachmann bundle pacing requires paced P-wave and/or electrogram criteria rather than anatomical septal orientation alone.^36^ A low-profile 12-lead electrocardiogram configuration positioned away from essential acoustic windows may facilitate these applications, although cable routing and sterile access require prospective evaluation.

### Study Limitations

Several limitations should be considered. First, this single-center study included 31 selected patients, representing 59.6% of the 52 consecutive patients screened within the Department of Structural Heart Disease. Approximately 4,000 de novo pacemaker implantations were performed hospital-wide during the same period, but most were managed by a separate arrhythmia center outside the clinical responsibility and approved research authorization of the study team. Those patients could not be prospectively assessed under this protocol. Consequently, the 52-patient screening cohort was not a hospital-wide consecutive sample, and the proportion of the broader institutional pacemaker population that might have met the TTE-only criteria is unknown. Eight department-referred patients were excluded because the required supine views could not be obtained; their mean body mass index was 24.5 ± 3.8 kg/m^2^. Although the age, body mass index, and pacing-indication distributions overlapped with those reported in some Chinese pacemaker cohorts,^37^, ^38^, ^39^ they differed from those reported in a contemporary European registry.^40^ This department-referred cohort was relatively young and lean and should be considered a highly selected feasibility population rather than representative of routine pacemaker practice. Patients with obesity, pulmonary hyperinflation, chest-wall deformity, unstable pacing-dependent atrioventricular block, or pregnancy were poorly represented or absent. Second, all enrolled patients met the mandatory supine acoustic-window criteria at 2 preprocedural assessments, and the protocol required assessment of all mandatory views during the procedure. However, the frequency, repetition, and duration of each view, changes in image quality during the procedure, and the frequency of transiently inadequate views were not prospectively recorded. Formal intraprocedural interobserver reproducibility metrics were also not collected. Third, lead location was defined using multiplane TTE criteria combined with acute electrical measurements but was not independently validated by fluoroscopy, computed tomography, or another reference method. TTE cannot continuously show the complete lead trajectory or fixation helix and cannot exclude an out-of-plane course; therefore, the reported locations are TTE-defined study endpoints, not definitive anatomical confirmation. The combined slack criteria were partly qualitative and were not independently validated as a substitute for fluoroscopic slack assessment. Fourth, all procedures were guided by the same experienced primary echocardiographer with real-time on-site review by a second echocardiographer, within a stable multidisciplinary team. This arrangement may limit reproducibility at centers with different staffing or experience. Fluoroscopic and temporary-pacing rescue remained immediately available, and costs, staffing requirements, and resource use were not formally evaluated. Fifth, the small sample size, absence of a control group, and exploratory team-level case-order analysis preclude conclusions regarding equivalence to conventional implantation, individual operator competence, or a generalizable training threshold. Larger controlled multicenter studies are required.

## Conclusions

In this selected cohort of patients with adequate supine acoustic windows, an experienced multidisciplinary team completed TTE-guided transvenous pacemaker implantation without fluoroscopy in 30 of 31 patients. Larger controlled multicenter studies involving more diverse patient populations and longer follow-up are needed to confirm its feasibility, safety, and long-term lead performance.

Perspectives **COMPETENCY IN MEDICAL KNOWLEDGE:** In selected patients with adequate prespecified supine acoustic windows, a coordinated multidisciplinary team may use multiwindow TTE to guide transvenous pacemaker implantation without fluoroscopy. TTE provides real-time assessment of lead-myocardial interaction and structural complications. This workflow requires a dedicated echocardiographer and immediate access to fluoroscopic and temporary-pacing rescue.

**TRANSLATIONAL OUTLOOK:** Controlled multicenter studies should include more diverse patients and higher-risk pacing indications, assess reproducibility across institutions and operators, compare clinical outcomes and resource use with conventional fluoroscopic implantation, and explore hybrid TTE/electroanatomical mapping and conduction-system pacing workflows.

## Funding support and author disclosures

This work was supported by the Beijing Research Ward Excellence Program (grant numbers BRWEP2024W014030100 and BRWEP2024W014030105). The authors have reported that they have no relationships relevant to the contents of this paper to disclose.

## Supplemental material

Video 1 Representative case 1 (9 minutes 41 seconds) showing stepwise TTE-guided dual-chamber implantation, including venous access, guidewire and sheath advancement, ventricular and atrial lead delivery/fixation, orthogonal imaging, slack assessment, and final surveillance. Representative case 2 (6 minutes 40 seconds) showing the same stepwise workflow and interpatient variability in obtainable TTE views and probe positioning.
