# Supplementary material for: Feasibility and Safety of Transthoracic Echocardiography–Guided Transvenous Permanent Pacemaker Implantation Without Fluoroscopy
Source: JACC Adv. 2026 Oct 5;5(11):103301. doi: 10.1016/j.jacadv.2026.103301 (PMC13673103; doi:10.1016/j.jacadv.2026.103301)
Supplement: Supplementary file 1 [file mmc2.docx]

**Supplementary Materials**

*Feasibility and Safety of Transthoracic Echocardiography–Guided Transvenous Permanent Pacemaker Implantation Without Fluoroscopy*

Table of Contents

[Figure S1. Exploratory team-level case-order analysis and regression diagnostics for total procedure time.	2](#_Toc236063033)

[Figure S2. Cumulative sum (CUSUM) analysis of procedural performance.	3](#_Toc236063034)

# **Figure S1.** Exploratory team-level case-order analysis and regression diagnostics for total procedure time.

**(A)** Scatter plot showing the association between chronological case order and total procedure time. Each point represents an individual case, and the red line represents the fitted unsegmented linear regression model. Procedure time decreased with increasing case order. **(B)** Residual-versus-fitted plot for the unsegmented ordinary least-squares regression model. The residuals were distributed around zero without an evident nonlinear pattern or marked heteroscedasticity.

These analyses describe a team-level chronological association and should not be interpreted as establishing individual operator proficiency or a training threshold.

# **Figure S2.** Cumulative sum (CUSUM) analysis of procedural performance.

(**A**) Binary CUSUM chart for acute procedural failure using a reference failure probability of p₀ = 0.10. The curve showed an early upward excursion following the single procedural failure and subsequently declined to −2.1 by case 31. Because no decision limits were prespecified, the analysis was interpreted descriptively and does not establish statistical process control or performance relative to an external benchmark. (**B**) Continuous CUSUM chart for total procedure time, calculated as the cumulative deviation from the overall mean procedure duration. The curve initially increased and subsequently declined, indicating that procedure times in later chronological cases were more often below the overall sample mean.

**Abbreviations:** CUSUM, cumulative sum.
